# Deubiquitinase USP8 inhibits HIV-1 replication by suppressing Tat-mediated viral transactivation

**DOI:** 10.1128/jvi.00598-26

**Published:** 2026-09-28

**Authors:** Jinsong Yuan, Weijing Yang, Mingxiu Xu, Haobo Hu, Shuhan Yang, Xiaodong Sun, Wenyan Zhang, Chen Huan

**Affiliations:** 1 Department of Infectious Diseases, Infectious Diseases and Pathogen Biology Center, The First Hospital of Jilin University 117971, Changchun, Jilin, China; 2 Institute of Virology and AIDS Research, The First Hospital of Jilin University 117971, Changchun, Jilin, China; 3 Department of Hepatobiliary and Pancreatic Surgery, General Surgery Center, The First Hospital of Jilin University 117971, Changchun, Jilin, China; 4 Key Laboratory of Organ Regeneration and Transplantation of The Ministry of Education, The First Hospital of Jilin University 117971, Changchun, Jilin, China; University Hospital Tübingen, Tübingen, Germany

**Keywords:** Tat, USP8, HIV-1, transcription, deubiquitination

## Abstract

Ubiquitin-specific proteases (USPs) modulate diverse virus-host interaction events through deubiquitination; thus, investigating their functional roles and molecular targets facilitates the interpretation of viral pathogenic mechanisms and informs the design of anti-virus strategies. The deubiquitinase USP8 has been reported to antagonize HIV-1 replication by reversing Vif-mediated ubiquitination of the host restriction factor A3G and shielding it from proteasomal degradation. Here, we identify an additional A3G-independent anti-HIV mechanism of USP8. In A3G-negative cells, USP8 exerts a potent inhibitory effect on HIV-1 replication by significantly suppressing viral transcription. Mechanistically, USP8 interacts with the HIV-1 trans-activator Tat through its C-terminal domain and mediates the removal of Tat’s polyubiquitination chains in a manner dependent on USP8 catalytic activity. This disrupts the association between Tat and the HIV-1 LTR transactivation response (TAR) element, thus impairing Tat-mediated viral transactivation. Furthermore, given the essential role of Tat in driving viral gene expression, silencing of USP8 triggers latent HIV-1 reactivation. Collectively, our results, along with previous work, reveal that USP8 acts as a compelling anti-HIV factor that, by regulating both host and viral proteins, ultimately suppresses HIV-1 replication. These results deepen our understanding of USP8-mediated HIV-1 restriction and provide insights for the development of novel anti-HIV therapeutic strategies.

**IMPORTANCE:**

Ubiquitination modification contributes a non-negligible part to the regulation of HIV-1 replication, and the interpretation of key factors involved in this process has significantly benefited our understanding of the virus-host interplay. USP8 is a ubiquitin-specific protease that was recently identified to restrict HIV-1 replication by stabilizing the host restriction factor A3G through reversing the Vif-mediated ubiquitination of A3G. Here, we demonstrate an additional A3G-independent anti-HIV mechanism of USP8. In A3G-negative cells, USP8 directly interacts with and deubiquitinates Tat to cleave its K63-linked ubiquitination, thereby suppressing Tat-mediated HIV-1 trans-activation. The suppression of Tat-driven viral transcription induced by USP8 requires the enzymatic activity of USP8 and is effective in different HIV-1 tropic strains. In addition, the fact that Tat is a novel substrate of USP8 is validated both in cells and by *in vitro* deubiquitination assays. These findings illustrate the dual regulatory roles of USP8 on both host antiviral factors and viral proteins, expanding our understanding of host-mediated HIV-1 restriction.

## INTRODUCTION

As an obligate intracellular pathogen, the life cycle of HIV-1 is substantially reliant on the dynamic interplay with the host regulatory machinery (1). Among these mechanisms, ubiquitination, the highly conserved post-translational modification process that plays crucial roles in nearly every aspect of eukaryotic cellular function (2), contributes a non-negligible part to the HIV-host interactions (3, 4). To establish the life cycle, both HIV-1 and the host have evolved strategies to exploit the cellular ubiquitination machinery for their respective benefits (5). For HIV-1, a typical example is the viral protein Vif, which hijacks host E3 ubiquitin ligases to mediate ubiquitination and proteasomal degradation of the host restriction factor APOBEC3G (A3G), thereby preventing A3G-induced hypermutation of the viral genome (6). In contrast, the host E3 ligase TRIM5α mediates ubiquitination of the HIV-1 capsid protein, blocking viral uncoating and reverse transcription, establishing a host defense mechanism against HIV-1 infection (7). Notably, ubiquitination is also a reversible process, in which ubiquitin conjugated to target substrates can be removed by deubiquitinating enzymes (DUBs), which thus act as key regulators of ubiquitination dynamics (8). In recent years, significant progress has been made in elucidating the roles of DUBs in modulating HIV-1 replication (9–11), which sheds light on the regulatory network of post-translational modifications in HIV-host interplay.

USP8 is a member of the ubiquitin-specific protease family, the largest subclass of DUBs (12). USP8 has been extensively investigated for its critical role in regulating a wide range of biological processes (13). For instance, USP8 contributes to maintaining embryonic stem cell stemness via deubiquitination of EPG5 (14). CHMP2B and Shrub/CHMP4, two key components of the ESCRT-III complex, have been shown to be substrates of USP8, and USP8-mediated deubiquitination plays an essential role in switching cell division (15). By deubiquitinating PD-L1, USP8 sustains the immunosuppressive microenvironment of tumors through upregulating PD-L1 levels (16). Thus, it can be concluded that by deubiquitinating specific substrate proteins, USP8 exerts profound impacts on cellular homeostasis and disease pathogenesis, and each identification of a novel substrate further deepens our comprehensive understanding of the regulatory processes and functions that USP8 is involved in. In our previous study, we found that USP8 counteracts Vif-induced ubiquitination of the antiviral protein A3G, thereby preserving A3G stability. This finding indicates that, in addition to the above-mentioned processes, USP8 also participates in regulating HIV-1 replication (10).

Of note, accumulating evidence has demonstrated that the modulation of a specific biological process by USPs may be achieved through the coordinated deubiquitination of multiple target proteins (17). Therefore, to investigate whether additional factors participate in the USP8-mediated HIV-1 restriction, we assessed the impact of USP8 on HIV-1 replication in A3G-negative cells. Our results reveal that USP8 retains a robust antiviral effect against HIV-1 in the absence of A3G, indicating a previously uncharacterized mechanism underlying its anti-HIV activity. Subsequent mechanistic investigations demonstrated that this antiviral function is reliant on the catalytic activity of USP8, and that the HIV-1 transactivator Tat acts as a substrate for it. USP8 interacts with Tat via its C-terminal domain and mediates the deubiquitination of Tat. Serving as the key determinant in driving HIV-1 transcription, Tat recruits the host positive transcriptional elongation factor b (P-TEFb) complex composed of cyclin T1 and cyclin-dependent kinase 9 (CDK9) to the TAR element of the HIV-1 LTR promoter, triggering efficient transcriptional elongation and viral replication (18). Notably, Tat is subject to extensive PTMs, whose dynamic regulation critically modulates its transactivating activity and thus viral gene expression. Our present study further extends this regulatory machinery to deubiquitination, identifying USP8 as a novel regulator that cleaves K63-linked polyubiquitination on Tat, thereby impairing Tat-mediated HIV-1 transactivation through attenuating its interaction with the TAR element. Moreover, this anti-HIV function shows broad-spectrum antiviral properties. Collectively, these observations underscore the dual role of USP8 in inhibiting HIV-1: on the one hand, USP8 deubiquitinates the host restriction factor A3G to exert antiviral effects, and on the other hand, it targets the viral transactivator Tat to suppress HIV-1 transcription. Taken together, our findings elucidate a novel regulatory mechanism governing the anti-HIV activity of USP8.

## RESULTS

### USP8 inhibits HIV-1 replication in A3G-negative cells

**Fig 1:**
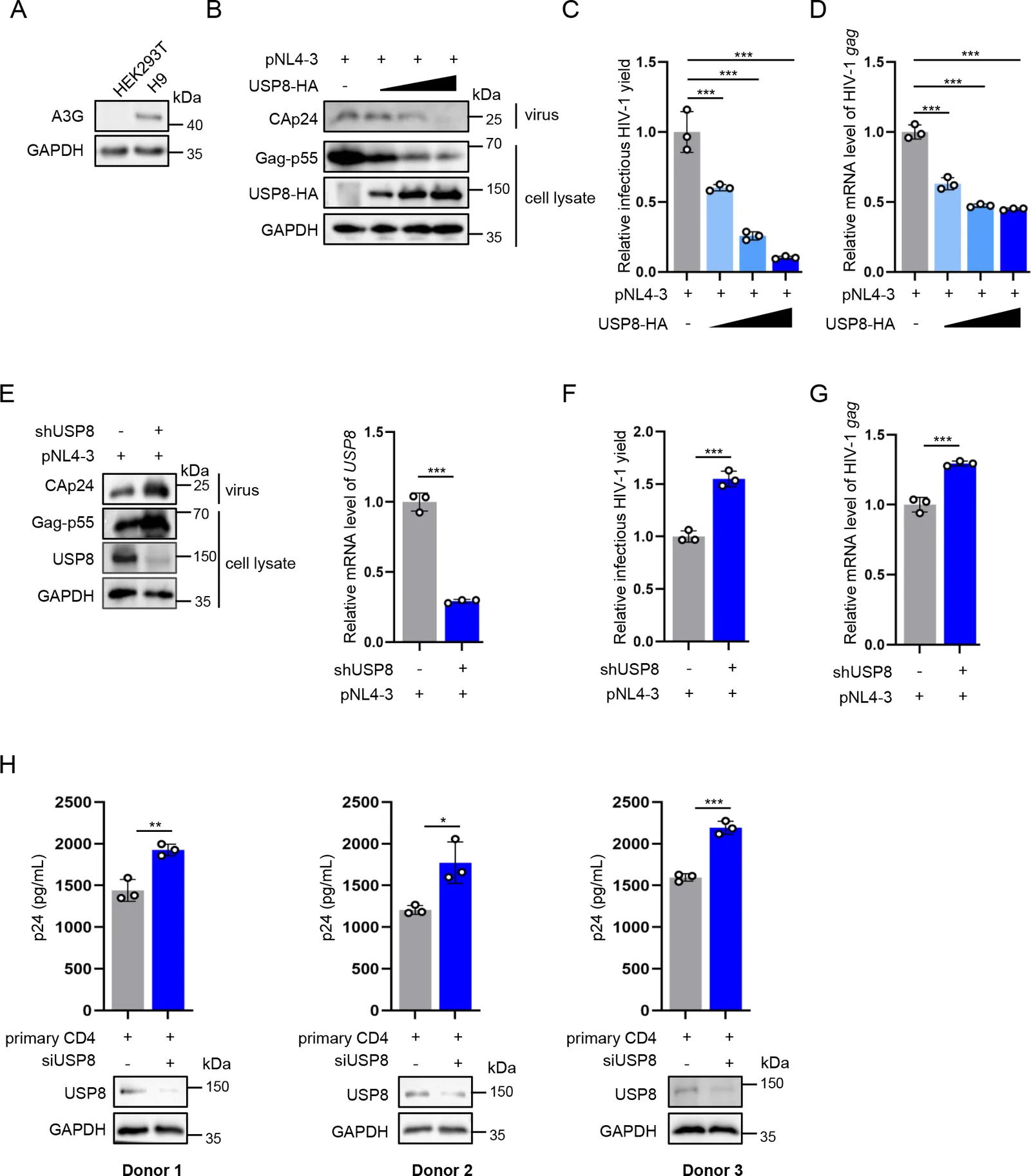
USP8 inhibits HIV-1 replication in A3G-negative cells. (**A**) H9 cells, which naturally express A3G, were used as a control, whereas HEK293T cells are A3G-negative. (**B–D**) USP8 inhibits HIV-1 replication in a dose-dependent manner in A3G-negative HEK293T cells by suppressing viral transcription. HEK293T cells were transfected with increasing doses of USP8-HA, together with the pNL4-3 viral vector. At 48 h post-transfection, viral supernatants and cells were collected, and the indicated protein levels were examined by immunoblotting (**B**). Then, the viral supernatants were used to infect TZM-bl cells for 48 h, and infectious virus production was quantified using the TZM-bl reporter cell infectivity assay (**C**). The mRNA level of HIV-1 *gag* was quantified by RT-qPCR, with the *gag* mRNA level in cells transfected with pNL4-3 plus the control vector normalized to 1 (**D**). (**E–G**) Silencing of USP8 enhances HIV-1 replication in HEK293T cells. The endogenous USP8 in HEK293T cells was knocked down using USP8-specific shRNA, and cells were subsequently transfected with pNL4-3. At 48 h post-transfection, levels of cellular Gag-p55 and supernatant CAp24 were examined by immunoblotting, and the knockdown efficiency of USP8 was also validated by RT-qPCR (**E**). Then, the infectious virus production was quantified (**F**), and the mRNA level of HIV-1 *gag* was measured (**G**). (**H**) Silencing of USP8 enhances HIV-1 replication in primary CD4+ T cells. CD4+ T cells isolated from three healthy donors were nucleofected with siRNA against USP8 and infected with HIV-1 NL4-3. HIV-1 yield in the supernatant was measured using a p24 ELISA assay. *P* values were calculated using ANOVA (**C and D**) or the two-tailed Student’s *t*-test (**F–H**). *P < 0.05, **P < 0.01, and ***P < 0.001.

To investigate whether USP8 regulates HIV-1 replication in an additional A3G-independent manner, pNL4-3 was co-transfected with increasing doses of USP8 into HEK293T cells, which lack endogenous A3G expression (Fig. 1A). At 48 h post-transfection, both cells and culture supernatants were collected and analyzed. The results revealed that, along with the USP8 amount increased, HIV-1 replication was significantly inhibited, as indicated by reduced levels of cellular Gag-p55 and supernatant CAp24 (Fig. 1B), as well as decreased infectious virus yield detected by TZM-bl reporter cells (Fig. 1C) and decreased viral mRNA levels examined using RT-quantitative PCR (qPCR) assays (Fig. 1D). These results implied that USP8 might exert its anti-HIV function through a mechanism independent of A3G. Conversely, we expanded and validated these observations from a loss-of-function perspective. As shown in Fig. 1E through G, knockdown of endogenous USP8 with shRNA potently enhanced HIV-1 replication, further supporting the role of USP8 in suppressing HIV-1 replication in A3G-negative cells. Notably, nucleofection of USP8-targeting short interfering RNA (siRNA) into primary CD4^+^ T cells replicated this phenotype (Fig. 1H), corroborating the above findings.

**Fig 2:**
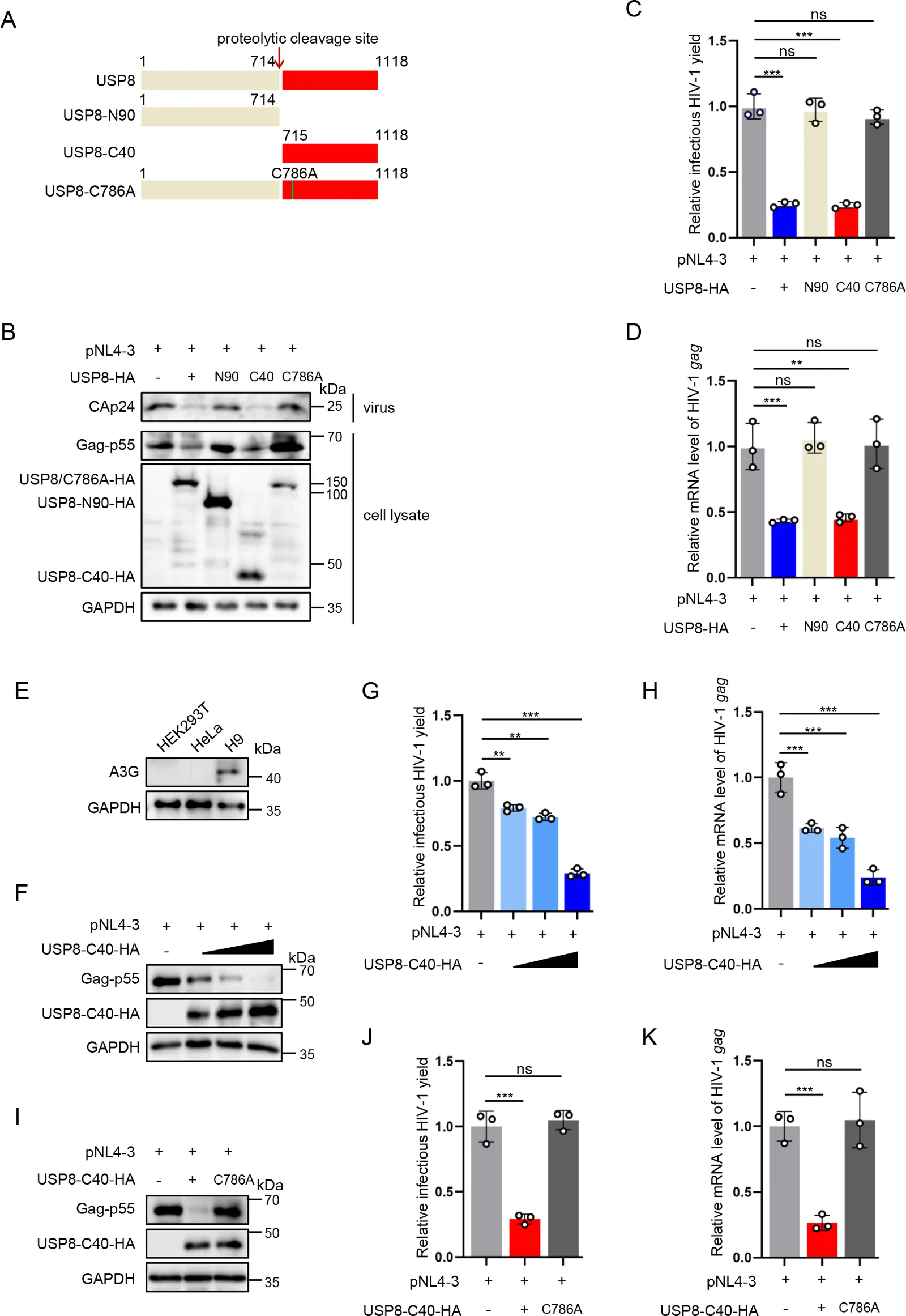
Deubiquitinating enzymatic activity is required for USP8 to restrict HIV-1 replication in A3G-negative cells. (**A**) The schematic illustrates USP8 WT, its truncations N90 and C40, and the catalytically deficient mutant C786A. The red arrow indicates the proteolytic cleavage site (p.714Arg). (**B–D**) Deubiquitinating enzymatic activity is required for USP8 to restrict HIV-1 replication in A3G-negative HEK293T cells. HEK293T cells were co-transfected with the pNL4-3 viral vector and USP8-HA, USP8-N90-HA, USP8-C40-HA, USP8-C786A-HA, or a control vector. At 48 h post-transfection, viral supernatants and cells were collected, and the indicated proteins were analyzed by immunoblotting (**B**). Viral supernatants were then used to infect TZM-bl cells for 48 h, and infectious virus production was quantified using the TZM-bl reporter cell infectivity assay (**C**). The mRNA level of HIV-1 *gag* was measured by RT-qPCR, with the *gag* mRNA level in cells transfected with pNL4-3 plus the control vector normalized to 1 (**D**). (**E–K**) USP8 inhibits HIV-1 replication in A3G-negative HeLa cells. (**E**) HeLa cells are A3G-negative. (**F–H**) USP8-C40 retains anti-HIV-1 activity. HeLa cells were co-transfected with increasing doses of USP8-C40, together with the pNL4-3 viral vector. At 48 h post-transfection, the level of cellular Gag-p55 was examined by immunoblotting (**F**). The viral supernatants were used to infect TZM-bl cells for 48 h, and infectious virus production was then quantified by measuring the luciferase activity (**G**). The mRNA level of HIV-1 *gag* was quantified by RT-qPCR, with cells transfected with pNL4-3 plus the control vector normalized to 1 (**H**). (**I–K**) The deubiquitinating enzymatic activity is required for USP8-C40 to restrict HIV-1 replication in HeLa cells. HeLa cells were co-transfected with the pNL4-3 viral vector plus USP8-C40, USP8-C40-C786A, or a control vector. *P* values were calculated using ANOVA. **P < 0.01, ***P < 0.001, and ns denotes no significance.

Next, we investigated whether deubiquitinating enzymatic activity is required for USP8 to restrict HIV-1 in A3G-negative cells. It has been reported that USP8 is processed through proteolytic cleavage at residue 714, resulting in an N-terminal 90-kDa fragment and a C-terminal 40-kDa fragment. The C-terminal 40-kDa fragment, which contains the residue C786 essential for enzymatic activity, retains catalytic function (19, 20). Therefore, we generated the C786A mutant lacking deubiquitinating activity, along with N-terminal (N90) and C-terminal (C40) fragments of USP8 (Fig. 2A), and compared their ability in restricting HIV-1 replication with that of wild-type (WT) USP8. As demonstrated in Fig. 2B through D, in A3G-negative HEK293T cells, USP8 significantly inhibits HIV-1 replication, consistent with the findings in Fig. 1B through D, and this restrictive effect was also observed in cells transfected with USP8-C40. However, the anti-HIV ability of USP8 was abolished when the enzymatic site was absent (N90) or mutant (C786A), indicating that deubiquitinating enzymatic activity is required for USP8 to restrict HIV-1 replication in A3G-negative cells. To further validate this hypothesis, another A3G-negative cell line HeLa (21) was employed (Fig. 2E); instead of using the full length USP8, its C-terminal (C40) was used because of the transfection efficiency. As shown in Fig. 2F through H, USP8-C40 dose-dependently inhibited HIV-1 replication in HeLa cells, and the enzymatic activity site C786 was required (Fig. 2I through K), demonstrating a consistent anti-HIV effect as observed in HEK293T cells.

Altogether, these results indicate that USP8 restricts HIV-1 replication through an alternative pathway that is independent of A3G, and this suppressive function requires its deubiquitinating enzymatic activity.

### USP8 suppressed Tat-mediated viral transactivation

**Fig 3:**
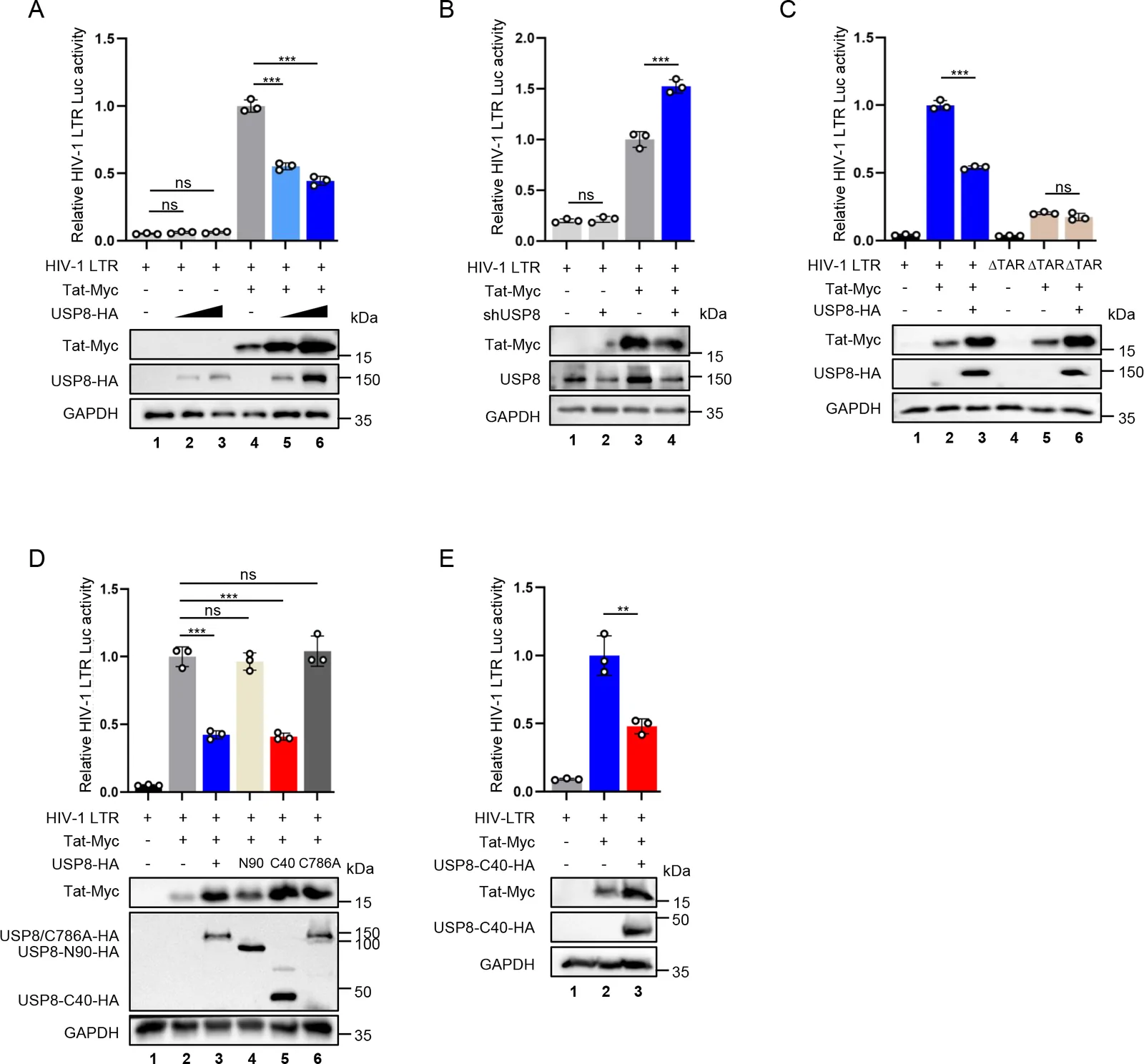
USP8 suppressed Tat-mediated viral transactivation. (**A and B**) USP8 suppresses HIV-1 LTR activity in a Tat-dependent manner. HEK293T cells were co-transfected with an HIV-1 LTR-luciferase construct and increasing amounts of USP8 in the absence or presence of Tat for 48 h, and LTR activity and protein levels were measured (**A**). Silencing USP8 enhanced HIV-1 LTR activity in HEK293T cells (**B**). (**C**) The TAR element is essential for USP8 to inhibit Tat-mediated transactivation. (**D**) USP8-C40 exerts anti-HIV-1 activity comparable to that of the WT. (**E**) The catalytic domain of USP8 (USP8-C40) suppresses Tat-mediated LTR transactivation in primary CD4+ T cells. The activity of cells transfected with HIV-1 LTR-luciferase plus Tat was normalized to 1. *P* values were calculated using ANOVA (**A and D**) or the two-tailed Student’s *t*-test (**B, C, and E**). **P < 0.01, ***P < 0.001, and ns denotes no significance.

Subsequently, we explored the underlying mechanism by which USP8 inhibits HIV-1 in A3G-negative cells. The downregulation of viral mRNA induced by USP8 overexpression (Fig. 1D and 2D) and promotion of viral mRNA levels by USP8 silencing (Fig. 1G) imply that USP8 may suppress viral transcription. It is now well recognized that HIV-1 LTR activity, especially its transactivation driven by the transactivator Tat, governs the efficiency of viral transcription (22, 23). Thus, we speculated that USP8 might regulate the LTR activity of HIV-1. To this end, an LTR-luciferase reporter plasmid was co-transfected with increasing amounts of USP8 in the absence or presence of Tat. As displayed in Fig. 3A, USP8 overexpression alone had no obvious effect on LTR activity (lanes 1–3). However, when Tat was co-expressed, USP8 dose-dependently reduced the Tat-mediated enhancement of LTR activity, despite the increased protein level of Tat induced by USP8 (Fig. 3A; lanes 4–6). In contrast, when endogenous USP8 was silenced, the LTR activity driven by Tat (Fig. 3B; lane 4 vs lane 3), rather than its basal activity (Fig. 3B; lane 2 vs lane 1), was further enhanced. These results collectively indicate that USP8 suppresses Tat-driven transactivation of HIV-1 transcription. To further confirm this, an LTR construct lacking the TAR element, the hairpin structure responsible for binding to Tat, was generated, and its activity under USP8 overexpression was evaluated. The results showed that deletion of the TAR region impaired Tat-induced activation of LTR activity (Fig. 3C; lane 5 vs lane 2), and USP8 no longer exhibited regulatory effects either (Fig. 3C; lane 6 vs lane 5), further confirming that Tat-mediated enhancement of LTR activity is required for USP8 to suppress HIV-1 transcription. Moreover, when further considering the domains and enzymatic activity of USP8, the results demonstrated that, consistent with its anti-HIV function, deubiquitinating enzymatic activity was essential for USP8 to regulate Tat-enhanced LTR activity. As shown in Fig. 3D, both the wild type and C-terminal fragment retaining catalytic function (C40) suppressed Tat-mediated transactivation (lanes 3 and 5 vs lane 2). By contrast, when the enzymatic site was deleted (N90) or mutated (C786A), the activity of LTR driven by Tat was not affected (Fig. 3D; lanes 4 and 6 vs lane 2). Moreover, we further validated these observations in primary CD4+ T cells and obtained consistent results showing that USP8-C40 significantly inhibits Tat-mediated LTR transactivation (Fig. 3E). Of note, when taking the levels of Tat into account, we found that despite the dysregulation of Tat’s transactivating function, USP8 did not reduce Tat abundance but rather increased it to a certain extent. Collectively, these results demonstrate that USP8 regulates both the abundance and the activity of Tat, and compared with the effect on increasing Tat levels, its suppression on HIV transcription is mainly attributed to the suppression of Tat-mediated transactivation.

### USP8 interacts with Tat via its C-terminal fragment

**Fig 4:**
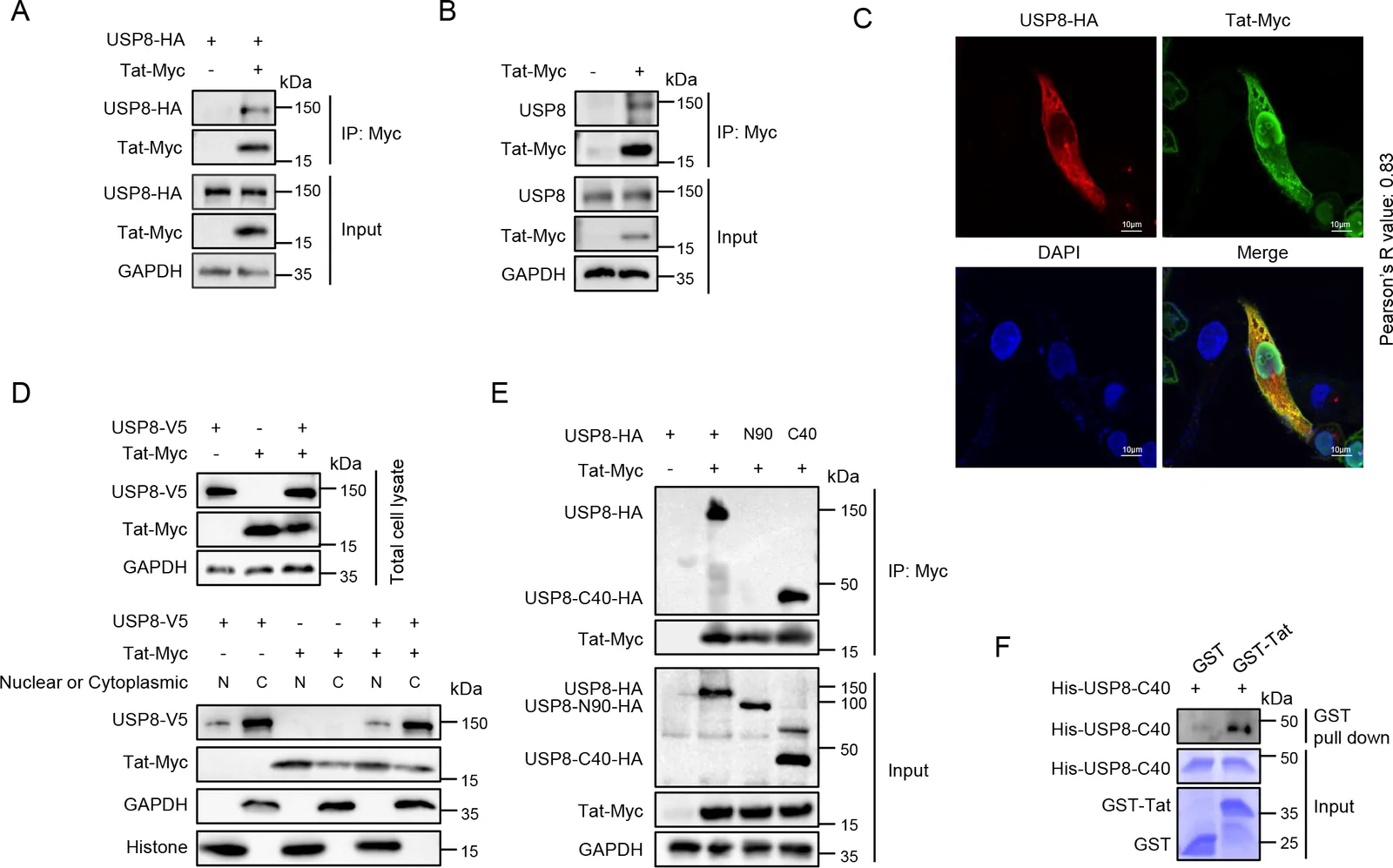
USP8 interacts with Tat via its C-terminal fragment. (**A and B**) Co-IP assays demonstrated the interaction between USP8 and Tat. HEK293T cells were transfected with USP8-HA together with Tat-Myc or a control vector. At 48 h post-transfection, cells were harvested and subjected to Myc IP followed by immunoblotting (**A**). In parallel, HEK293T cells transfected with Tat-Myc or a control vector were subjected to Myc IP, followed by immunoblotting with an antibody against USP8 (**B**). (**C**) USP8 co-localizes with Tat. HeLa cells were co-transfected with USP8-HA and Tat-Myc. An anti-HA antibody was used to immunostain USP8-HA (red), Tat-Myc was detected using an anti-Myc antibody (green), and nuclei were stained with DAPI (blue). Images were acquired using a Nikon laser-scanning confocal microscope. Scale bar, 10 µm. The Pearson’s correlation coefficient calculated using ImageJ was 0.83. (**D**) The nuclear-cytoplasm separation assay was performed on HEK293T cells transfected with USP8 and Tat. (**E**) The USP8-C40 interacts with Tat. HEK293T cells were co-transfected with expression vectors as indicated. At 48 h post-transfection, cells were harvested and subjected to Myc IP followed by immunoblotting. (**F**) USP8-C40 binds efficiently to GST-Tat *in vitro*. GST pull-down assays were performed using GST-Tat or a GST control together with His-USP8-C40, and GST-Tat efficiently pulled down His-USP8-C40.

Having confirmed the regulation of USP8 on Tat’s transactivating function, we reasoned that this regulatory effect may be mediated through a potential interaction between USP8 and Tat. To better assess this issue, Co-IP experiments were performed, and the results showed that when using Tat-Myc as a bait, both ectopically expressed (Fig. 4A) and endogenous USP8 (Fig. 4B) were effectively co-immunoprecipitated. In addition, confocal assays showed that USP8 and Tat co-localize in the cytoplasm with a Pearson’s *R* value = 0.83 (Fig. 4C), further confirming the interaction between USP8 and Tat. Moreover, the nuclear-cytoplasm separation assay additionally verified the distribution of both USP8 and Tat (Fig. 4D). To further define the USP8 domains involved in its interaction with Tat, we detected the Tat-binding capabilities of the C40 and N90 fragments of USP8, and found that the C-terminal domain (USP8-C40) was sufficient to bind Tat, whereas the N-terminal domain (N90) lacks this ability (Fig. 4E), in line with their distinction in regulating HIV-1 replication and Tat-enhanced LTR activity. Based on these observations, we further investigated the direct interaction between Tat and USP8-C40. GST-Tat and His-USP8-C40 were expressed and purified *in vitro*, then GST or GST-Tat was incubated with His-USP8-C40. The GST pulldown assay revealed that, in comparison to GST alone, GST-Tat effectively binds USP8-C40 (Fig. 4F). Taken together, USP8 directly interacts with Tat via its C-terminal fragment.

### USP8 deubiquitinates Tat

**Fig 5:**
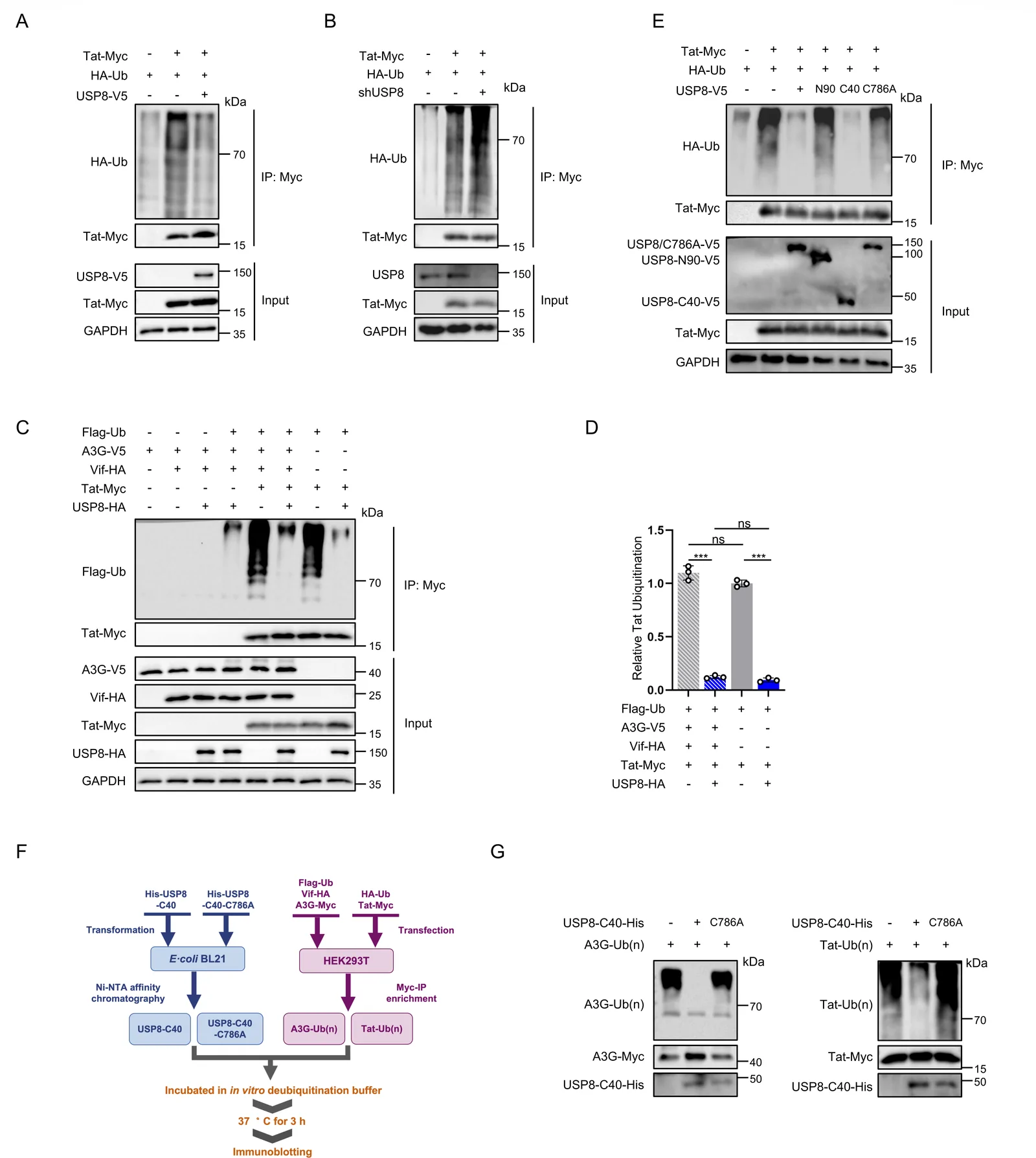
USP8 deubiquitinates Tat. (**A and B**) USP8 downregulates Tat ubiquitination. HEK293T cells were co-transfected with Tat-Myc and either USP8-V5 or a control vector, together with HA-Ub. At 48 h post-transfection, cells were harvested and subjected to Myc IP followed by immunoblotting (**A**). The same assay was performed in HEK293T cells with endogenous USP8 knockdown (shUSP8), in which silencing USP8 resulted in elevated Tat ubiquitination (**B**). (**C and D**) USP8 retains its deubiquitinating function toward Tat in the presence of Vif and A3G. HEK293T cells were co-transfected with the expression vectors as indicated. At 36 h post-transfection, MG132 (10 μM) was added to block proteasomal degradation. At 48 h post-transfection, cells were harvested and subjected to Myc IP followed by immunoblotting (**C**). Quantification shows the relative ubiquitin levels, with cells transfected with Ub and Tat normalized to 1 (**D**). (**E**) The USP8 C-terminal was sufficient to remove ubiquitin chains from Tat. HEK293T cells were co-transfected with Tat-Myc and HA-Ub together with USP8-V5, USP8-N90-V5, USP8-C40-V5, USP8-C786A-V5, or a control vector. At 48 h post-transfection, cells were harvested for Myc IP and immunoblotting. (**F**) The schematic illustrates the workflow of the *in vitro* deubiquitination assay. E*. coli* BL21 cells were transformed with the indicated plasmids, and USP8-C40 and its catalytic mutant USP8-C40-C786A were purified by Ni-NTA affinity chromatography. HEK293T cells were transfected with the indicated plasmids, and the ubiquitinated substrates A3G-Ub(n) and Tat-Ub(n) were enriched by Myc-IP. USP8-C40 or USP8-C40-C786A was then incubated with the ubiquitinated substrates, followed by immunoblotting analysis. (**G**) USP8-C40 removes ubiquitin chains from Tat *in vitro*. USP8-C40 or USP8-C40-C786A was incubated with ubiquitinated Tat in an *in vitro* deubiquitination buffer at 37 °C for 3 h, with ubiquitinated A3G included as a positive control substrate, and the reaction products were subsequently analyzed by immunoblotting. *P* values were calculated using two-tailed Student’s *t*-test. ***P < 0.001 and ns denotes no significance.

Based on these observations, we speculated that Tat may be a new substrate modulated by USP8. To examine this hypothesis, we investigated whether Tat’s polyubiquitination was affected by USP8. As shown in Fig. 5A, upon USP8 overexpression, polyubiquitination of Tat was significantly reduced. By contrast, stable knockdown of USP8 enhanced Tat’s ubiquitination (Fig. 5B). Subsequently, we examined whether USP8 could exert the deubiquitinating function on Tat when it devotes to reverse the Vif-mediated ubiquitination of A3G. As shown in Fig. 5C and D, upon the overexpression of A3G and Vif, the deubiquitination of Tat induced by USP8 was not affected, indicating that USP8 deubiquitinates Tat independently of the A3G/Vif signaling axis. Moreover, when further assessing the functional domains and enzymatic activity site (Fig. 5E), *in vivo* deubiquitination assays revealed that when the C-terminal was deleted (USP8-N90), or harboring a deficient enzymatic activity site (USP8-C786A), USP8 lost the ability to cleave Tat’s ubiquitination. Notably, the C40 fragment of USP8 was found to be sufficient to deubiquitinate Tat. To further confirm these observations, we *in vitro* expressed and purified USP8-C40, as well as its catalytically deficient mutant USP8-C40-C786A, and investigated their ability to deubiquitinate Tat. As shown in Fig. 5F, polyubiquitinated Tat was enriched using Myc antibody-conjugated beads; in parallel, polyubiquitinated A3G was also enriched and served as a control, and then polyubiquitinated Tat or A3G was incubated with purified USP8-C40 or USP8-C40-C786A in deubiquitination buffer. Immunoblotting assays revealed that incubation with USP8-C40, rather than USP8-C40-C786A, significantly reduced the ubiquitin chains on A3G (left panel of Fig. 5G), reflecting the efficiency of the assays we performed. Moreover, the ubiquitination of Tat was notably cleaved by USP8-C40 as well (right panel of Fig. 5G). These results collectively indicate that Tat is a new substrate of USP8.

### Cleavage of K63-linked polyubiquitination on Tat elucidates the mechanism underlying USP8-mediated inhibition of LTR activity

**Fig 6:**
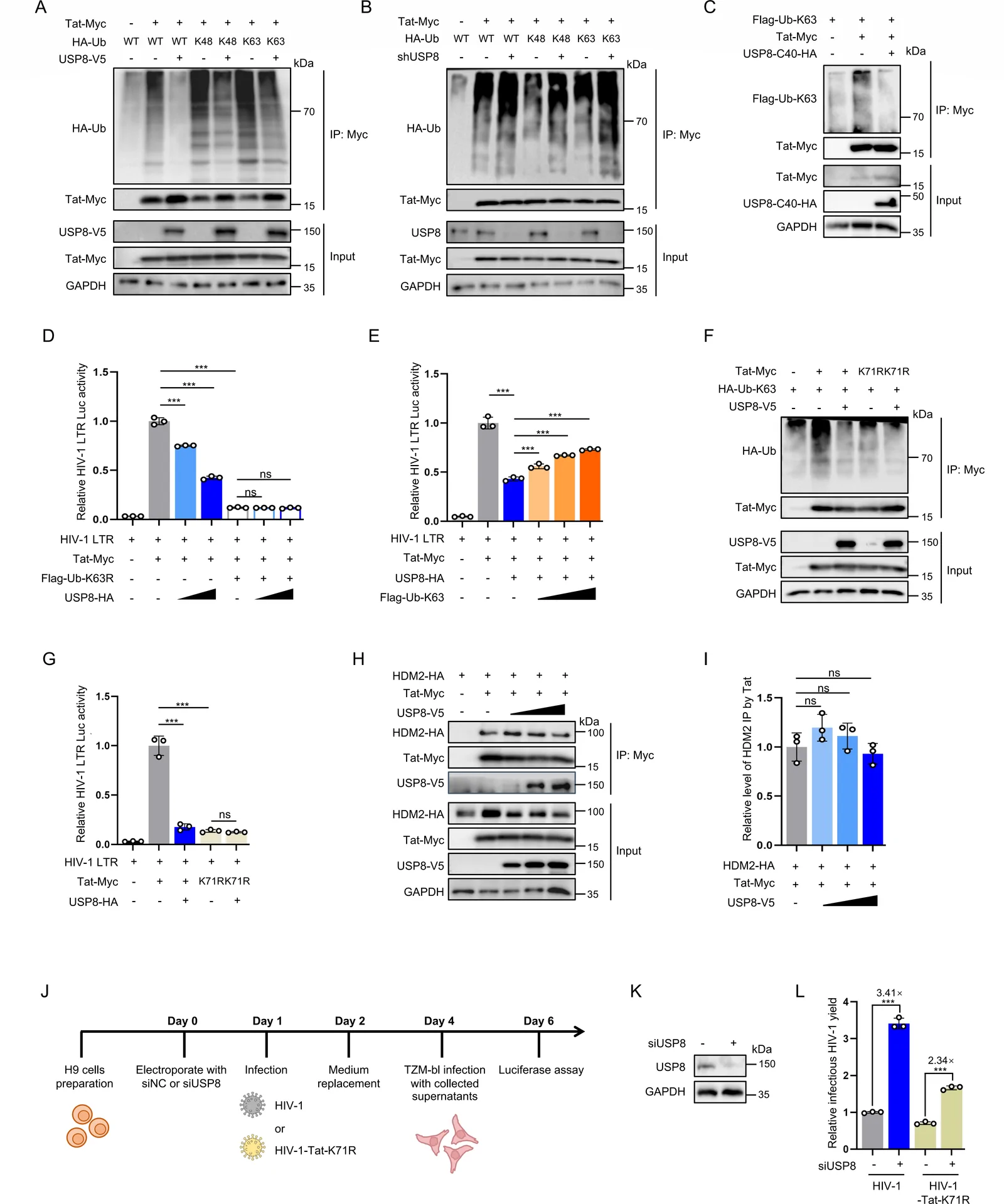
Cleavage of K63-linked polyubiquitination on Tat elucidates the mechanism underlying USP8-mediated inhibition of LTR activity. (**A and B**) USP8 removes K48- and K63-linked ubiquitin chains from Tat. HEK293T cells were co-transfected with the expression vectors as indicated. At 48 h post-transfection, cells were harvested and subjected to Myc IP followed by immunoblotting. (**C**) USP8-C40 removes K63-linked polyubiquitination of Tat in primary CD4^+^ T cells. (**D**) USP8-mediated inhibition of LTR activity driven by Tat was abolished upon Ub-K63R overexpression. HEK293T cells were co-transfected with the expression vectors as indicated. At 48 h post-transfection, the LTR activity and protein levels were measured, with the activity of cells transfected with HIV-1 LTR-luciferase plus Tat normalized to 1. (**E**) Ub-K63 rescues USP8-mediated LTR suppression. HEK293T cells were co-transfected with the expression vectors as indicated. The assay is described in panel D. (**F and G**) USP8 removes K63-linked ubiquitin chains from Tat mainly at K71. K63-linked ubiquitination at K71 is critical for Tat-mediated transactivation of HIV-1 LTR. The K71R mutation markedly decreased K63-linked ubiquitination of Tat and impaired its LTR transactivation activity, whereas USP8 had little effect on the ubiquitination level or transactivation activity of Tat-K71R. HEK293T cells were co-transfected with the expression vectors as indicated. At 48 h post-transfection, cells were harvested and subjected to Myc IP followed by immunoblotting (**F**), and the LTR activity was measured, with the activity of cells transfected with HIV-1 LTR-luciferase plus Tat normalized to 1 (**G**). (**H and I**) USP8 does not alter the interaction between Tat and HDM2. HEK293T cells were co-transfected with the expression vectors as indicated. At 48 h post-transfection, cells were harvested and subjected to Myc IP followed by immunoblotting (**H**). Quantification shows the relative level of HDM2 IP by Tat, with cells transfected with HDM2, Tat, plus the control vector normalized to 1 (**I**). (**J–L**) Tat-K71R mutation attenuates the effect of USP8 knockdown on HIV-1 replication in H9 cells. (**J**) The schematic illustrates the workflow of the H9 infection experiment. (**K**) The H9 cells nucleofected with siRNA targeting USP8 were detected using an immunoblotting assay, were packaged in HEK293T cells, and virus normalized to equal p24 levels was used to infect H9 cells transfected with control siRNA or USP8-targeting siRNA. (**L**) HIV-1 replication of wild type and Tat K71R mutant NL4-3 virus in control or USP8-silencing H9 cells was quantified using the TZM-bl reporter cell assay. *P* values were calculated using ANOVA (**D, E, and I**) and the two-tailed Student’s *t*-test (**G and L**). ***P < 0.001 and ns denotes no significance.

Previous studies have reported that Tat undergoes both K48-linked and K63-linked ubiquitination, which was implicated in regulating Tat’s stability (24, 25) and functional activity (26), respectively. Therefore, we further detected the ubiquitin chain type of Tat cleaved by USP8. As shown in Fig. 6A and B, both the K48-linked and K63-linked ubiquitination of Tat were decreased by USP8, and when endogenous USP8 was knocked down, K48-linked, especially the K63-linked, ubiquitination of Tat potently increased. These observations may explain the above-mentioned observation that USP8 enhances Tat stability yet simultaneously impairs its transactivating function, and the attenuation of transactivating function outweighs the improvement of Tat abundance. Given the established role of K63-linked polyubiquitination in regulating Tat’s transactivity and its marked reduction by USP8, we next focused on the K63-linked polyubiquitination of Tat. First, we performed the Co-IP assays on primary CD4+ T cells to confirm USP8-mediated regulation of Tat ubiquitination and found that overexpression of the catalytic domain of USP8 (USP8-C40) significantly reduced the K63-linked polyubiquitination of Tat in primary CD4+ T cells (Fig. 6C). Subsequently, we further investigated the requirement of K63-linked polyubiquitination in the USP8-mediated suppression of LTR activity, a ubiquitin K63R mutant (Ub-K63R) was generated and employed in luciferase reporter assays. As shown in Fig. 6D, overexpression of Ub-K63R potently attenuated Tat-mediated LTR transactivation and abrogated USP8’s ability to inhibit LTR activity. In contrast, when USP8 expression was fixed and increasing doses of wild-type Ub-K63 were added to the system, USP8-mediated LTR suppression was effectively rescued (Fig. 6E), confirming that USP8 exerts its inhibitory effect on Tat-transactivated LTR activity in a K63-linked polyubiquitination-dependent manner. Moreover, the K71 residue of Tat has been identified as the specific acceptor site for K63-linked polyubiquitination, which was catalyzed by the E3 ubiquitin ligase HDM2 (26). We therefore generated the K71R mutant of Tat and assessed the effect of USP8 on its ubiquitination. As shown in Fig. 6F, Tat K71R displayed a marked reduction in K63-linked polyubiquitination and was resistant to USP8-mediated deubiquitination. Importantly, a luciferase reporter assay revealed that USP8-mediated suppression of LTR activity was abolished when Tat harboring the K71R mutant (Fig. 6G). Notably, USP8 did not alter the interaction between Tat and HDM2 (Fig. 6H and I). These findings collectively indicate that cleavage of K63-linked polyubiquitination on Tat elucidates the mechanism underlying USP8-mediated inhibition of LTR activity.

Building on these observations, we further established a functional connection between this antiviral pathway and USP8-dependent regulation of A3G. To this end, the A3G-expressing H9 cells were employed to compare the replication of wild-type and NL4-3 viruses containing the Tat-K71R mutation (HIV-1-Tat-K71R) upon USP8 silencing (Fig. 6J). The endogenous USP8 in H9 cells was efficiently knocked down using a Nucleofection assay (Fig. 6K), and subsequently infected with HIV-1 or HIV-1-Tat-K71R virus with equivalent input p24 levels. As shown in Fig. 6L, HIV-1 NL4-3 carrying the Tat K71R mutant exhibited attenuated viral replication compared to wild-type NL4-3 (lane 3 vs lane 1), and when endogenous USP8 was knocked down, the replication of both wild type (lane 2 vs lane 1) and HIV-1-Tat-K71R (lane 4 vs lane 3) was enhanced, with a milder upregulation detected in the HIV-1-Tat-K71R group. These results indicate that USP8-mediated regulation of Tat and A3G function cooperatively rather than competitively to restrict HIV-1 replication, with parallel antiviral effects from the two regulatory pathways.

### USP8 impairs the interaction between Tat and the TAR element in the HIV-1 LTR

**Fig 7:**
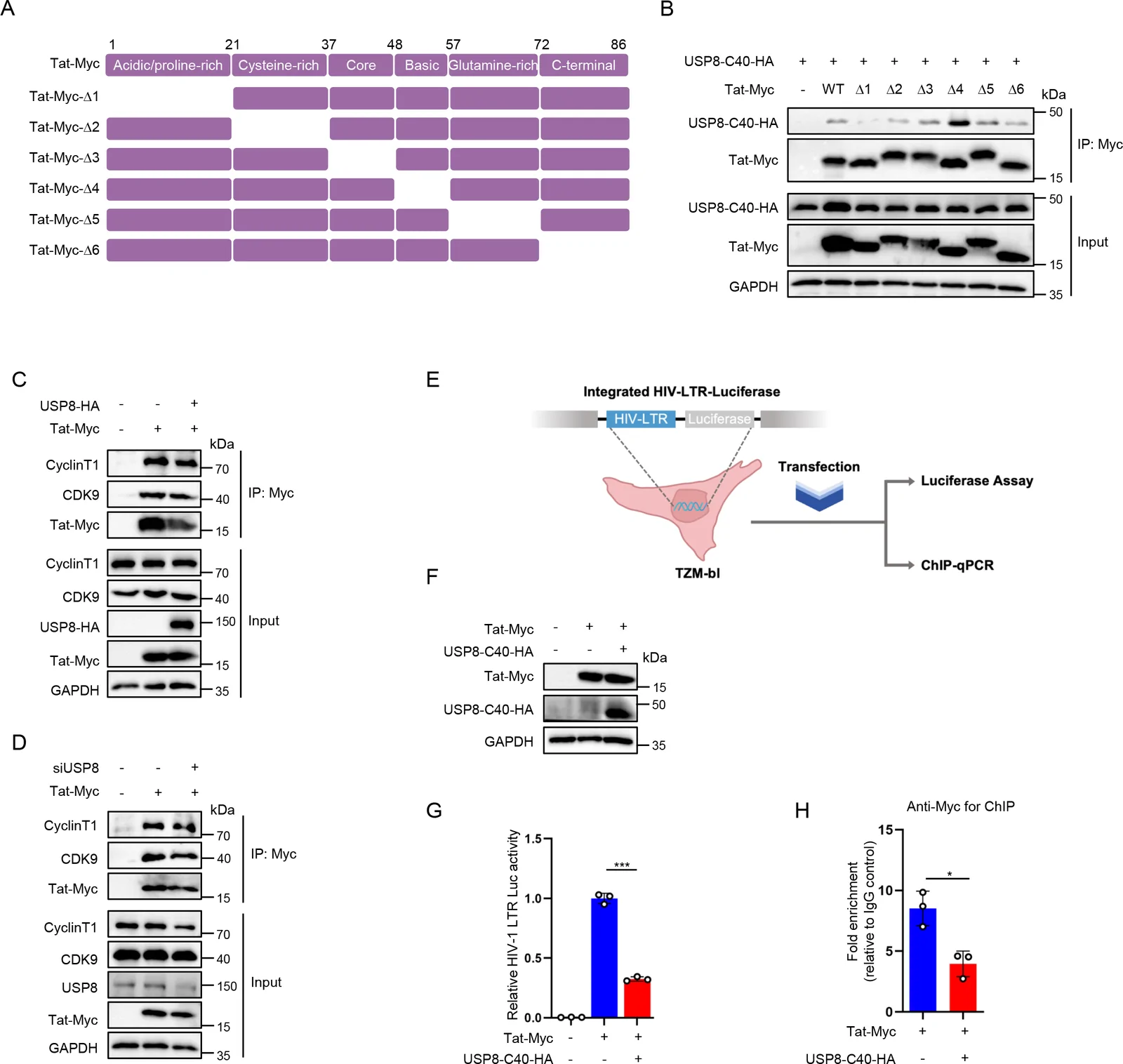
USP8 impairs the interaction between Tat and the AR element in the HIV-1 LTR. (**A**) Schematic diagram of Tat domains and truncated mutants. (**B**) Tat lacking the acidic/proline-rich domain (Tat-△1) exhibits impaired binding to USP8. (**C and D**) USP8 does not interfere with Tat-mediated recruitment of the P-TEFb complex. (**E–H**) USP8 disrupts the interaction between Tat and the TAR element in the HIV-1 LTR. The schematic illustrates the experimental design using TZM-bl cells, which stably harbor a luciferase reporter driven by HIV-LTR, to detect the effect of USP8 on Tat-TAR interaction (**E**). Following transfection with the indicated plasmids, a portion of the cells were subjected to immunoblotting (**F**) and luciferase assay (**G**), and other cells were subjected to ChIP assays with anti-Myc antibody (IgG as negative control), followed by qPCR using primers targeting the TAR region (**H**). *P* values were calculated using the two-tailed Student’s *t*-test. *P < 0.05 and ***P < 0.001.

HIV-1 Tat consists of six functional domains, which act in a collaborative manner to trigger efficient viral transcription (27). Therefore, to gain insight into the detailed mechanism of USP8-mediated regulation of Tat, we constructed truncated mutants of Tat lacking each of these six domains (Fig. 7A), and detected their interaction with USP8. Compared with wild-type Tat, only the mutant lacking Acidic/proline-rich domain (Tat-△1) displayed a weakened ability to bind USP8 (Fig. 7B). Given that the Acidic/proline-rich domain of Tat mediates the initial binding to cyclin T1, and subsequently displaces the inhibitory 7SK snRNP from P-TEFb (27), we next explored whether USP8 affects the recruitment of the P-TEFb complex by Tat. Co-IP assays revealed that USP8 overexpression (Fig. 7C) or silencing (Fig. 7D) exerted no effect on the abundance of cyclin T1 and CDK9 associated with Tat. These results rule out the possibility that USP8 disrupts Tat-mediated hijacking of the P-TEFb complex, implying that USP8 most likely interacts with Tat after P-TEFb dissociates from 7SK snRNP. Subsequently, we examined whether the Tat-TAR interaction was affected by USP8. TZM-bl cells, which harbor an integrated LTR driving the expression of firefly luciferase, were employed to evaluate TAR enriched by Tat upon the modulation of USP8 (Fig. 7E). Tat and USP8-C40 were overexpressed (Fig. 7F), and consistent with the observations in HEK293T cells, LTR activity triggered by Tat was significantly suppressed by USP8-C40 (Fig. 7G). Notably, chromatin immunoprecipitation (ChIP)-qPCR assays revealed that TAR region enriched by the Tat was reduced upon the co-transfection of USP8-C40 (Fig. 7H). Altogether, these results demonstrate that USP8 inhibits Tat transactivation by impairing the interaction between Tat and the TAR element in the HIV-1 LTR.

### The inhibitory effect of USP8 on Tat-mediated transactivation is broad spectrum

**Fig 8:**
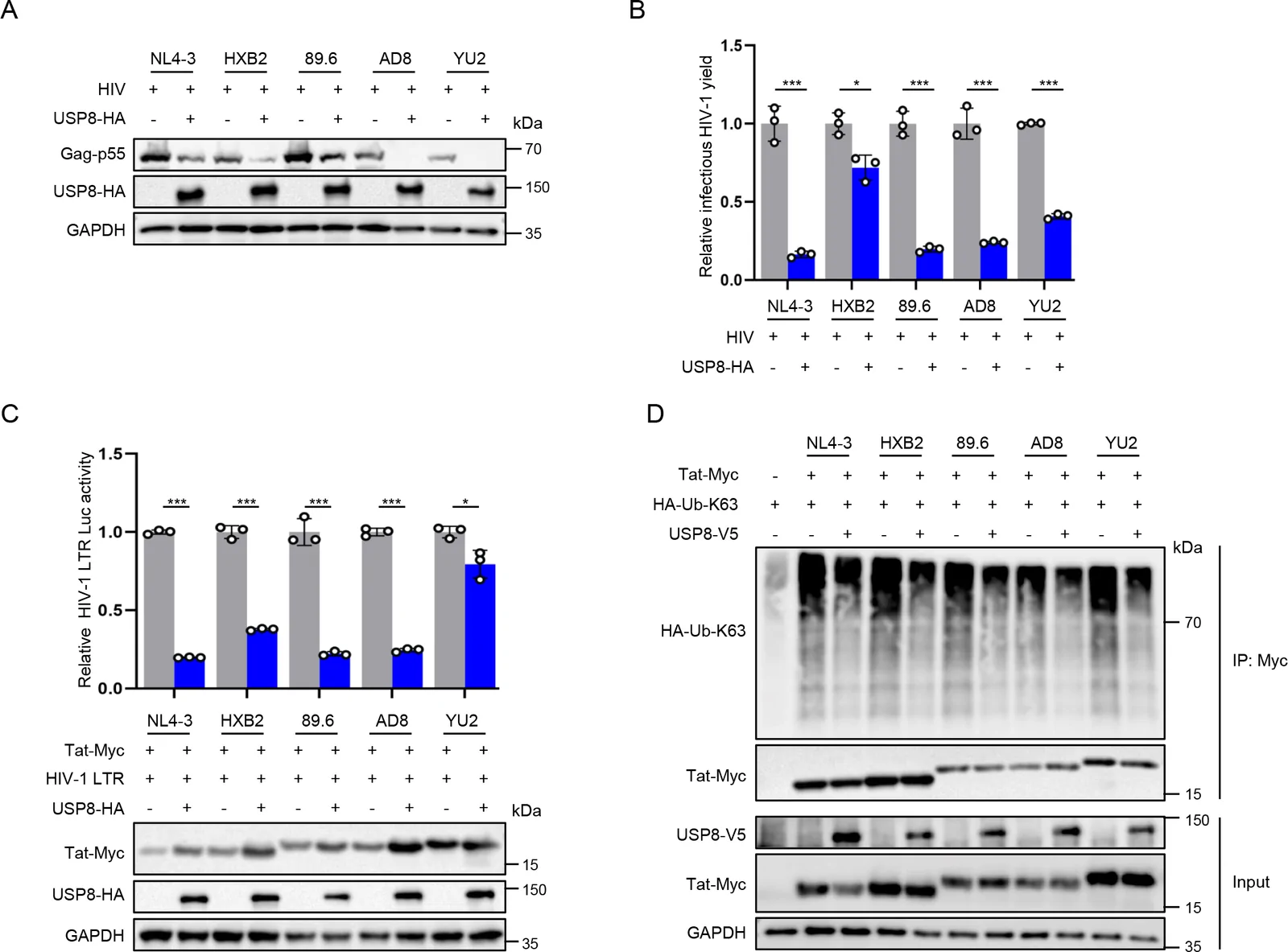
The inhibitory effect of USP8 on Tat-mediated transactivation is broad spectrum. (**A and B**) USP8 inhibits the replication of HIV-1 strains of different tropisms. HEK293T cells were co-transfected with HIV-1 viral vectors representing strains of different tropisms (CXCR4-tropic: NL4-3 and HXB2, dual-tropic/R5 X 4-tropic: 89.6, CCR5-tropic: AD8 and YU2) with USP8 or a control vector. At 48 h post-transfection, cells were harvested, and the indicated protein levels were analyzed by immunoblotting (**A**). The viral supernatants were used to infect TZM-bl cells for 48 h, and infectious virus production was then quantified by measuring the luciferase activity, with the activity of cells transfected with the corresponding HIV-1 plus control vector normalized to 1 (**B**). (**C**) USP8 broadly suppresses LTR activation mediated by Tat proteins from different HIV-1 tropic strains. HEK293T cells were co-transfected with an HIV-1 LTR-luciferase construct and Tat in the presence or absence of USP8 for 48 h. LTR activity and protein expression levels were then measured, with the activity of cells transfected with corresponding HIV-1 Tat plus control vector normalized to 1. (**D**) USP8 removes K63-linked ubiquitin chains from Tat derived from strains of different tropisms. HEK293T cells were co-transfected with the expression vectors as indicated. At 48 h post-transfection, cells were harvested and subjected to Myc IP by immunoblotting. *P* values were calculated using the two-tailed Student’s *t*-test. *P < 0.05, ***P < 0.001, and ns denotes no significance.

To further determine whether the antiviral effect of USP8 is broad spectrum, we transfected different tropic HIV-1 strains (CXCR4-tropic: NL4-3 and HXB2, dual-tropic/R5 X 4-tropic: 89.6, CCR5-tropic: AD8 and YU2) into HEK293T cells in the presence or absence of USP8. The results showed that USP8 reduced the level of cellular Gag-p55 across these different tropic HIV-1 strains (Fig. 8A) and effectively inhibited the production of infectious viral particles (Fig. 8B). We next examined the effect of USP8 on the transcriptional activation of LTR driven by different tropic Tat proteins, as well as on their ubiquitination levels. As shown in Fig. 8C and D, USP8 exerted inhibitory effects on Tat-mediated transcription and efficiently reduced ubiquitination of different tropic Tat proteins. Taken together, the inhibitory effect of USP8 on Tat-mediated transactivation exhibits a broad-spectrum antiviral activity.

### USP8 enhances HIV-1 latency

**Fig 9:**
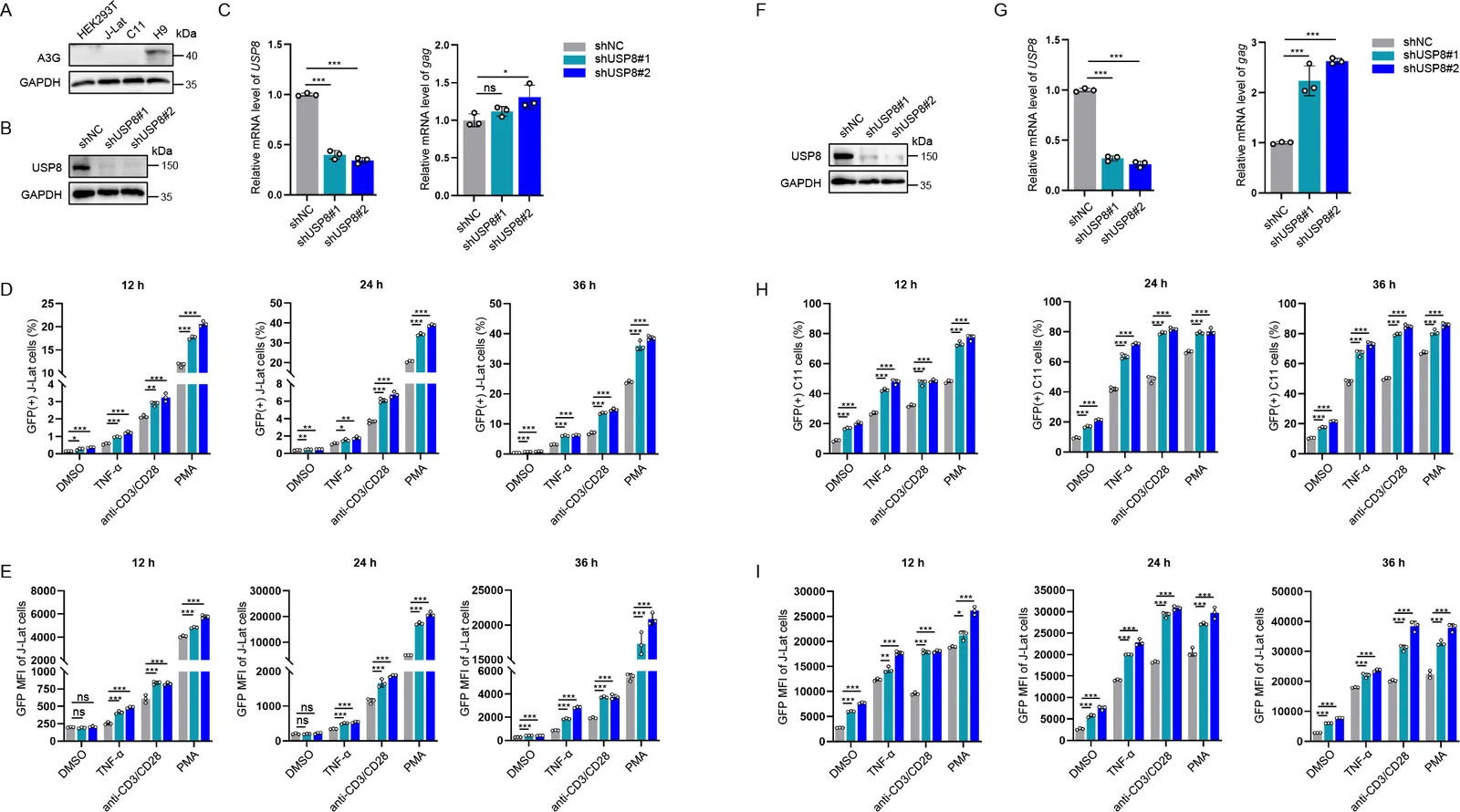
USP8 enhances HIV-1 latency. (**A**) HIV latent J-Lat and C11 cells are A3G-negative. (**B**) Endogenous USP8 in J-Lat cells was knocked down using two USP8-specific shRNAs, and the knockdown efficiency was assessed by immunoblotting assay. (**C**) The mRNA level of *USP8* and HIV-1 *gag* in control and shUSP8 J-Lat cells was quantified by RT-qPCR, with J-Lat-shNC cells normalized to 1. (**D and E**) USP8 silencing activates latent HIV-1 replication in J-Lat cells. Control and shUSP8 J-Lat cells were treated with TNF-α (20 ng/mL), anti-CD3/CD28 Dynabeads (2 μL per 1 × 10⁵ cells), or PMA (1 μM) for 12, 24, or 36 h, and DMSO served as the control. GFP-positive cells were detected by flow cytometry. The percentage of GFP-positive cells (**D**) and the GFP MFI (**E**) were quantified using FlowJo. (**F–I**) USP8 silencing activates latent HIV-1 replication in C11 cells. Assays were performed as described in panels B–E. *P* values were calculated using ANOVA. **P < 0.05, **P < 0.01, ***P < 0.001, and ns denotes no significance.

Given the significant suppression of viral transcription by USP8, we speculated that USP8 may contribute to the maintenance of HIV-1 latency, a process governed by viral transcription (28). To better elucidate this issue, we took advantage of Jurkat-based HIV-1 latent cell lines J-Lat 6.3 (29) and C11 (30), both of which express GFP upon reactivation. First, we confirmed the absence of A3G in J-Lat 6.3 and C11 cells (Fig. 9A). The lentivirus containing shRNA targeting two different regions of USP8 was packaged to knock down the endogenous USP8 in J-Lat 6.3 cells (Fig. 9B), and USP8 silencing resulted in mild enhanced viral transcription levels (Fig. 9C). Under the time-course treatment with multiple latency-reversing stimuli, including TNF-α, anti-CD3/CD28, and PMA, USP8 silencing resulted in pronounced reactivation of latent HIV-1, as evidenced by increased percentages (Fig. 9D; Fig. S1) and mean fluorescence intensity (MFI, Fig. 9E) of GFP-positive cells. In addition, similar results were also obtained in C11 cells (Fig. 9F through I; Fig. S2). These observations indicate that USP8 silencing relieves the transcriptional repression of latent HIV-1 genomes to promote viral reactivation in an A3G-independent manner.

## DISCUSSION

Protein ubiquitination is a precisely regulated and reversible post-translational process that participates in nearly every aspect of eukaryotic cellular function (2, 31), and the arms race between HIV-1 and the host is also modulated by this machinery (3, 32). Clarifying the key factors and underlying mechanisms of this regulatory procedure has significantly enhanced our understanding of the pathogenesis of HIV infection, and importantly, has provided insights for anti-HIV therapy design (33, 34). As a deubiquitinase involved in diverse biological processes, USP8, as demonstrated in our previous work, also contributes to the host restriction of HIV-1 through cleaving Vif-mediated polyubiquitination of the host factor A3G (10). In this study, we identify an alternative anti-HIV-1 mechanism of USP8 that is independent of its previously reported regulation of A3G. We demonstrate that USP8 directly targets Tat, the transactivator of HIV-1, and mediates the cleavage of its K63-linked polyubiquitination at the K71 residue. This deubiquitination impairs its transactivating activity toward the HIV-1 LTR promoter by disrupting Tat-TAR interaction, thereby potently suppressing viral transcriptional activation. These findings uncover a previously unrecognized substrate of USP8 and provide new evidence for its role in regulating viral replication.

As a key viral accessory protein, Tat is indispensable for HIV-1 transcriptional activation. Though merely 86 or 101 amino acids in length, Tat undergoes diverse PTMs that confer substantial complexity to its regulatory activity, including ubiquitination (24, 35), phosphorylation (36, 37), and acetylation (38). Recently, two USPs, USP21 and USP45, have been reported to target Tat and suppress HIV-1 transcription through distinct mechanisms. USP21 restricts viral replication not only by deubiquitinating Tat to promote its proteolytic degradation but also by epigenetically repressing cyclin T1 transcription, which further destabilizes Tat protein (39). By contrast, USP45 functions as an interferon-stimulated DUB that specifically removes K48- and K63-linked ubiquitin chains from Tat at lysine 19, accelerating Tat turnover and restraining transcription to sustain HIV-1 latency (40). Here, we show that USP8 is a new regulator of Tat. Notably, our findings demonstrate that USP8 attenuates both K48- and K63-linked polyubiquitination of Tat. Intriguingly, when the K63-linked ubiquitination of Tat was disrupted by introducing either Ub-K63R or Tat-K71R, a residue that acts as the acceptor site for K63-linked polyubiquitination of Tat, the regulation of USP8 on Tat-mediated transactivation was impeded, revealing that the regulatory activity of Tat is more functionally critical than its protein abundance in mediating the downstream biological processes of HIV-1. These findings confirm that K63-linked polyubiquitination is a positive regulatory modification for Tat activity, and USP8 exerts its anti-HIV-1 effect by reversing this PTM. Our identification of USP8 as a host deubiquitinase that specifically regulates Tat K63-linked polyubiquitination enriches the understanding of how host cells modulate viral protein function through PTM manipulation.

**Fig 10:**
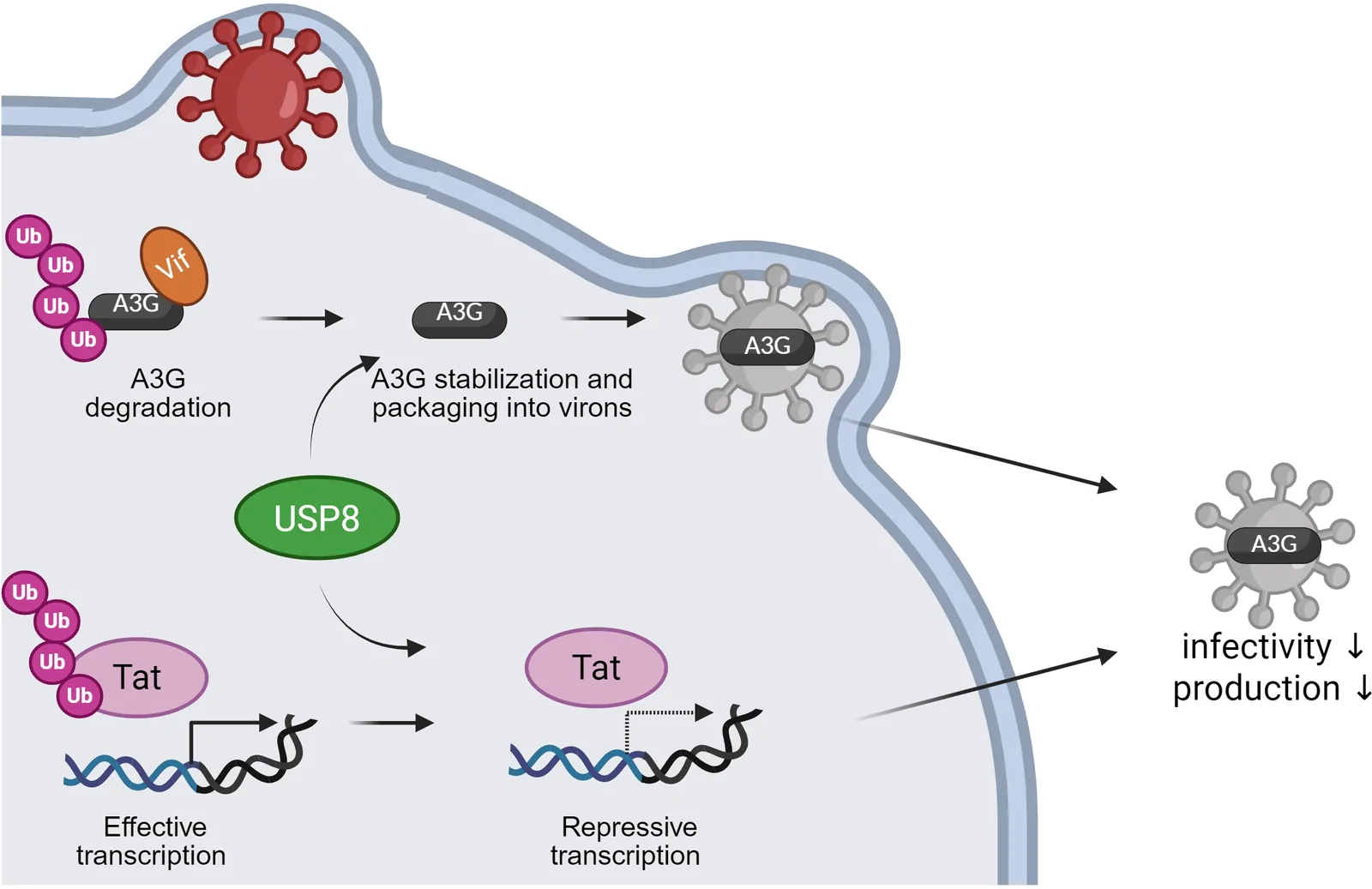
Schematic of the molecular mechanisms underlying the dual anti-HIV function of USP8. USP8 acts as an anti-HIV factor that regulates both host and viral proteins, and ultimately suppresses HIV-1 replication. On one hand, USP8 stabilizes the host restriction factor A3G by antagonizing Vif-mediated ubiquitination and proteasomal degradation, preserving cellular A3G levels and facilitating A3G incorporation into progeny virions, leading to impaired virion infectivity. On the other hand, USP8 targets the viral protein Tat and interferes with viral transcription by deubiquitinating Tat, reducing viral production. This figure was generated using BioRender (http://biorender.com/).

Furthermore, the identification of Tat as a novel substrate of USP8 advances our mechanistic understanding of USP8 anti-HIV activity. In addition to the established mechanism that USP8 maintains steady-state levels of host restriction factor A3G, which impairs viral infectivity by introducing hypermutations into the HIV-1 genome (41, 42), the present study reveals that USP8 suppresses HIV-1 transactivation through deubiquitinating Tat (Fig. 10). Importantly, several lines of evidence support the notion that these two antiviral branches function in parallel, rather than competing with each other. First, USP8-mediated Tat deubiquitination remains unaffected by A3G (Fig. 5C). Second, the antiviral potency of USP8 is attenuated against HIV-1 carrying the Tat K71R mutation (Fig. 6L), which disrupts the lysine 71 residue of Tat essential for USP8-mediated regulation, compared with the virus expressing wild-type Tat. Collectively, our findings establish USP8 as a pivotal factor in antagonizing HIV-1 replication through multiple regulatory mechanisms.

## MATERIALS AND METHODS

### Plasmid construction

The USP8, USP8-N90, and USP8-C40 were constructed by PCR amplification from USP8-HA/Flag (#79639; Addgene, Watertown, USA) and then cloned into VR1012 at the *Sal*I and *BamH*I sites of a C-terminal HA tag. V5-tagged USP8, USP8-N90, and USP8-C40 were generated by PCR amplification from their corresponding HA-tagged plasmids. The NL4-3-Tat-K71R, Tat-K71R-Myc, USP8-C786A-HA/V5, USP8-C40-C786A-HA, and USP8-C40-C786A-pET28a were generated from their corresponding wild-type plasmids by site-directed mutagenesis. Primers required for PCR are listed in Table 1. The HDM2 expression plasmid was purchased from Addgene. The Tat-Myc (43), USP8-C40-pET28a (10), Vif-HA (44), A3G-Myc (44), A3G-V5 (45), Renilla (46), HIV-1-LTR-luciferase, and HIV-1-LTR-∆TAR-luciferase (47) were described before. Flag-Ub, Flag-Ub-K63, Flag-Ub-K63R, HA-Ub, HA-Ub-K48, HA-Ub-K63, HIV-1 viral plasmids, and Tat from different tropic strains expression vectors were constructed and maintained in our laboratory.

**TABLE 1:** Primers used for plasmid construction and RT-qPCR

Primer name	Primer direction	Sequence (5′−3′)
USP8/C40-HA to V5-F	Forward	CTGATGTAGCCACAGGTAAGCCTATCCCTAACCCTCTCCTCGGTCTCGACTCTACGTAAATCGCGGCCG
USP8/C40-HA to V5-R	Reverse	CTGTGGCTACATCAGTTACTCGTGG
USP8-N90-HA to V5-F	Forward	CTGGATCCAGATCTGGTAAGCCTATCCCTAACCCTCTCCTCGGTCTCGACTCTACGTGAAGATCTGCTGT
USP8-N90-HA to V5-R	Reverse	CAGATCTGGATCCAGGCGCCTGGTC
USP8-C786A-F	Forward	GTAACTTAGGAAATACTGCATATATGAACTCAATATTG
USP8-C786A-R	Reverse	TGCAGTATTTCCTAAGTTACGAAGTCCAG
Tat-K71R-F	Forward	AGCGTCCCTGTCTAGGCAGCCTACATCTCA
Tat-K71R-R	Reverse	TAGACAGGGACGCTTGGTGTGTC
RT-Gag-F	Forward	GTGTGGAAAATCTCTAGCAGTGG
RT-Gag-R	Reverse	CGCTCTCGCACCCATCTC
RT-USP8-F	Forward	CTGAAAGACTCTCTGAAAGCCT
RT-USP8-R	Reverse	CCTTTCTCTTTGGTCTCACAT
RT-GAPDH-F	Forward	GCAAATTCCATGGCACCGT
RT-GAPDH-R	Reverse	TCGCCCCACTTGATTTTGG
TAR-F	Forward	AGCTTTCTACAAGGGACTTTCCGC
TAR-R	Reverse	ATTGAGGCTTAAGCAGTGGGTTCC

### Stable cell line construction

Two USP8-specific shRNAs (shUSP8#1: 5′-CCGGTAAGAGTTATGTGCACAGTGCCCTCGAGGGCACTGTGCACATAACTCTTTTTTTG-3′, shUSP8#2: 5′-CCGGCAGTATACAACAAATGCTACTCGAGTAGCATTTGTTGTATACTGTTTTTG-3′) were cloned into the lentiviral vector pLKO.1-puro (#8453; Addgene). HEK293T cells were co-transfected with shUSP8-pLKO.1 or control pLKO.1 together with the packaging plasmids psPAX2 and pMD2.G. At 48 h post-transfection, the culture supernatants containing packaged lentivirus were harvested and used to infect HEK293T, J-Lat 6.3, or C11 cells. Stable cell lines were generated by selection in medium containing puromycin (3 μg/mL) for 7 days.

### Cell culture and transfection

HEK293T (catalog no. CRL-11268), HeLa (catalog no. CCL-2), and TZM-bl (catalog no. PTA-5659) were cultured in Dulbecco’s modified Eagle’s medium (DMEM; #C11995500BT, Thermo, Grand Island, USA) supplemented with 10% fetal bovine serum (FBS; #ST30-3302, PAN Seratech, Aidenbach, Germany). The peripheral blood mononuclear cells (PBMCs) were isolated from whole blood by Ficoll density gradient centrifugation, and the CD4+ T cells were subsequently purified from PBMCs using anti-CD4 microbeads (#130-045-101, Miltenyi Biotec, Germany) according to the manufacturer’s instructions. The HIV-1 latent cell line C11 was a kind gift from H. Z. Zhu (The College of Life Science, Fudan University). H9 (catalog no. HTB-176), J-Lat 6.3 (catalog no. 9846), C11, and primary CD4+ T cells were cultured in RPMI 1640 medium supplemented with 10% FBS. All cell lines were cultured at 37°C in a humidified atmosphere containing 5% CO_2_.

HEK293T cells were seeded in 6-well plates 12 h before transfection and transfected at approximately 70% confluence using polyethyleneimine (PEI; #23966, Polysciences, Warrington, PA, USA) according to the manufacturer’s instructions. For each well, the total amount of plasmid DNA was kept constant by adding the corresponding control vector. The amounts of plasmids used were as follows: HIV-1 viral plasmids, 200 ng; USP8-HA/V5, 600 ng; USP8-N90-HA/V5, 200 ng; USP8-C40-HA/V5, 200 ng; USP8-C786A-HA/V5, 600 ng; HIV-LTR/∆TAR, 200 ng; Tats, 100 ng; Renilla, 2 ng; Ub expression plasmids, 600 ng; Vif-HA, 100 ng; A3G-V5, 200 ng; and HDM2-HA, 400 ng. For dose-dependent experiments, increasing amounts of USP8-HA/V5 were used at 200, 400, and 600 ng or 300 and 600 ng; Flag-Ub-K63 was used at 200, 400, and 600 ng. HeLa and TZM-bl cells were seeded in 6-well plates and transfected at approximately 50% confluence using Lipofectamine 3000 in combination with the P3000 reagent (#L3000-008, Thermo) according to the manufacturer’s instructions. For each well, the total amount of plasmid DNA was kept constant by adding the corresponding control vector. The amounts of plasmids used were as follows: NL4-3, 300 ng; USP8-C40/-C786A-HA, 600 ng. For dose-dependent experiments, increasing amounts of USP8-C40-HA were used at 200, 400, and 600 ng. The primary CD4+ T cells and H9 cells were nucleofected using the P3 Primary Cell 4D-Nucleofector X Kit (#V4XP-3024, Lonza, Basel, Switzerland) with program FI-115 according to the manufacturer’s instructions. Primary CD4^+^ T cells and H9 cells were electroporated with the indicated siRNAs at a final concentration of 200 nM.

### Immunoblotting analysis

Cells were harvested and lysed in 1× SDS loading buffer (0.08 M Tris [pH 6.8] containing 2.0% SDS, 10% glycerol, 0.1 M dithiothreitol, and 0.2% bromophenol blue) and boiled at 100°C for 15 min. The proteins were separated on 12% SDS-PAGE gels and transferred onto polyvinylidene fluoride membranes for immunoblotting. The membranes were incubated with primary antibodies, washed three times with TBST (20 mM Tris, 150 mM NaCl, and 0.1% Tween-20) for 10 min each, and then incubated with the corresponding horseradish peroxidase (HRP)-conjugated secondary antibody (#115-035-062, #111-035-045, Jackson) according to the manufacturer’s instructions. The membranes were washed again three times with TBST for 10 min each and then visualized using a multi-functional imaging system.

The following antibodies were used in this assay: rabbit polyclonal anti-USP8 (#A7031, ABclonal, Wuhan, China), rabbit polyclonal anti-APOBEC3G (#D221663, Sangon Biotech, Shanghai, China), rabbit polyclonal anti-CDK9 (#ab76320, Abcam, Cambridge, UK), rabbit polyclonal anti-cyclin T1 (#ab184703, Abcam), rabbit polyclonal anti-Myc (#16286-1-AP, Proteintech, Chicago, IL, USA), rabbit polyclonal anti-HA (#715500, Thermo), mouse monoclonal anti-Myc (#05-724, Millipore), mouse monoclonal anti-HA (#901514, Biolegend, San Diego, CA, USA), mouse monoclonal anti-His (#D110002-0100, BBI, Shanghai, China), mouse monoclonal anti-V5 (#R960-25, Invitrogen, Carlsbad, CA, USA), mouse monoclonal anti-Flag (#F1804, Sigma, St. Louis, MO, USA), mouse monoclonal anti-Histone (#PMK121W, BioPMK, Wuhan, China), mouse monoclonal anti-GAPDH (#RM2002, Beijing Ray Antibody Biotech, Beijing, China), and mouse monoclonal anti-Gag-p24 (#1513, NIH AIDS Reagents, USA).

### Luciferase assay

TZM-bl cells, which are derived from HeLa cells, contain an integrated HIV-1 LTR promoter-driven luciferase reporter gene. The supernatants from HEK293T or HeLa cells transfected with HIV-1 viral vectors were collected, and a volume of 60 μL was used to infect TZM-bl cells. TZM-bl cells were harvested 48 h after infection for further analysis. For the dual-luciferase reporter assays, HEK293T cells were transfected with HIV-1-LTR or HIV-1 LTR-∆TAR reporter plasmid together with a Renilla luciferase reporter plasmid as an internal normalization control. The assays were performed using the Dual-Luciferase Reporter Assay System (#E1910, Promega) according to the manufacturer’s instructions, and luminescence was measured with a GloMax 20/20 luminometer.

### RNA extraction and RT-qPCR

Total RNA was extracted using the RNA Total Extraction Kit (#YY101, Epzyme Biotech, Shanghai, China) according to the manufacturer’s instructions. To eliminate potential plasmid-derived DNA contamination, the extracted RNA was subsequently treated with RQ1 RNase-free DNase (#M6101, Promega, Madison, USA). The cDNA was synthesized from the purified RNA using the EasyScript First-Strand cDNA Synthesis SuperMix (#AE301, TransGen Biotech, Beijing, China) according to the manufacturer’s instructions. RT-qPCR was performed on a Roche LightCycler 480 system using the MonAmp ChemoHS qPCR Mix (#MQ00401S, Monad, Suzhou, China). Gene expression levels were normalized to GAPDH, and relative changes were determined using the 2^−ΔΔCt^ method. Primer sequences used in this study are listed in Table 1.

### Co-IP

For Co-IP assays examining the interaction of USP8, USP8-N90, USP8-C40, or HDM2 with Tat, the indicated plasmids were co-transfected into HEK293T cells. For endogenous immunoprecipitation, HEK293T cells were transfected with Tat-Myc or a negative control vector. For immunoprecipitation of ubiquitinated Tat, the indicated plasmids were co-transfected into HEK293T cells. At 36 h post-transfection, MG132 (10 µM) was added to prevent proteasomal degradation. At 48 h post-transfection, cells were harvested and washed twice with cold PBS, and then centrifuged at 10,000 × *g* for 30 min at 4°C. The cells were resuspended in lysis buffer (PBS containing 1% Triton X-100 and a complete protease inhibitor cocktail [Roche]) and lysed by sonication. The cell lysates were centrifuged at 10,000 × *g* for 30 min at 4°C, and the supernatants were incubated with agarose beads conjugated to the specified antibody overnight at 4°C. The reaction mixtures were washed six times with wash buffer (20 mM Tris, pH 7.5, 100 mM NaCl, 0.1 mM EDTA, and 0.05% Tween-20) and subsequently analyzed by immunoblotting.

### Confocal microscopy

The cellular localization of Tat and USP8 was detected by confocal microscopy. HeLa cells were transfected with Tat-Myc and USP8-HA. At 36 h post-transfection, cells were digested with trypsin and transferred into glass-bottom dishes (#BDD011035, JETBIOFIL, Guangzhou, China) containing culture medium supplemented with MG132 to prevent proteasomal degradation and incubated for 12 h to avoid excessive cell aggregation. The cells were then fixed in 4% paraformaldehyde at room temperature for 5 min and washed three times with PBS. After washing, the cells were permeabilized with 0.1% Triton X-100 for 10 min and washed again with PBS. Subsequently, the cells were blocked with 5% BSA in PBS at room temperature for 1 h to prevent nonspecific binding. The cells were then incubated with mouse monoclonal anti-Myc and rabbit polyclonal anti-HA antibodies. Nuclear staining was performed using DAPI (#D9542, Sigma), and the cells were incubated at room temperature for 1 h. Finally, the cells were washed three times with PBS for 5 min each and analyzed by a Nikon laser scanning confocal microscope.

The following antibodies were used in this assay: mouse monoclonal anti-Myc (#05-724, Millipore), rabbit polyclonal anti-HA (#715500, Thermo), Alexa Fluor 488 (#A11001, Thermo), and Alexa Fluor 568 (#A11011, Thermo).

### Nuclear-cytoplasm separation

HEK293T cells were collected 48 h post-transfection and washed twice with cold PBS. Cell pellets were obtained by centrifugation at 12,000 × *g* for 10 min at 4°C and resuspended in 400 μL of hypotonic lysis buffer (10 mM HEPES, pH 8.0; 1.5 mM MgCl₂; 10 mM KCl; and 0.5 mM DTT). After incubation at 4°C for 30 min with gentle rotation, NP-40 was added to a final concentration of 0.5% (vol/vol), followed by vigorous vortexing for 1 min. The lysates were centrifuged at 7,000 × *g* for 10 min at 4°C to separate the cytoplasmic and nuclear fractions. The supernatants were collected as cytoplasmic extracts, whereas the nuclear pellets were washed 4–5 times with cold PBS to minimize cytoplasmic contamination. Both fractions were subjected to immunoblotting analysis.

### ChIP-qPCR

Chromatin immunoprecipitation assays were performed using a ChIP Kit (#26157, Thermo) according to the manufacturer’s instructions. Briefly, TZM-bl cells were transfected with the indicated plasmids for 48 h and then cross-linked with 1% formaldehyde at room temperature for 10 min. After cross-linking, chromatin was prepared and immunoprecipitated with an anti-Myc antibody or IgG control, and the immunoprecipitated DNA was purified and analyzed by qPCR using primers spanning the TAR region of the HIV-1 LTR. Primer sequences are listed in Table 1.

### Infection and reactivation

For HIV-1 infection assays, 5.0 × 10⁶ primary CD4^+^ T cells or H9 cells were nucleofected and cultured for 24 h prior to infection. Primary CD4^+^ T cells were infected with HIV-1, whereas H9 cells were infected with either HIV-1 WT or HIV-1 Tat-K71R virus, each at 2 ng CAp24 equivalent. Twenty-four hours later, the culture medium was replaced with fresh medium, and the cells were cultured for an additional 48 h. For primary CD4^+^ T cells, viral production was quantified by measuring p24 levels in the culture supernatants using an ELISA assay. For H9 cells, culture supernatants containing progeny viruses were collected and used to infect TZM-bl cells, and the production of infectious virions was measured using luciferase activity.

HIV-1 latency reactivation was evaluated by flow cytometry to determine the effect of USP8 on HIV-1 latency. J-Lat/C11-pLKO.1 cells and J-Lat/C11-shUSP8#1, #2 cells were reactivated with TNF-α (20 ng/mL), anti-CD3/CD28 Dynabeads (2 μL per 1 × 10⁵ cells), or PMA (1 μM) for 12, 24, or 36 h. Cells treated with DMSO served as the negative control. Following reactivation, the percentage of GFP-positive cells was analyzed by flow cytometry.

### Protein purification

6×His-USP8-C40-pET28a and 6×His-USP8-C40-C786A-pET28a were expressed in the BL21(DE3) and purified using Ni-NTA agarose. The eluted proteins were supplemented with 5% glycerol and stored at −80°C for subsequent experiments. The GST and GST-Tat fusion proteins were obtained from the Institute of Virology and AIDS Research, First Hospital of Jilin University.

### GST affinity isolation

GST or GST-Tat fusion proteins were incubated with His-USP8-C40 in GST pull-down buffer (10 mM Tris-HCl, pH 8.0; 1 mM EDTA; 100 mM NaCl; freshly added protease inhibitor cocktail) supplemented with GST affinity purification resin (#C600031-0010, Sangon Biotech) at 4°C for 12 h. After incubation, the beads were washed six times with cold PBS, and the bound proteins were subsequently analyzed by western blotting.

### *In vitro* deubiquitination assay

Ubiquitinated A3G and Tat were isolated from HEK293T cells. For A3G ubiquitination, cells were co-transfected with Flag-Ub, A3G-Myc, and Vif-HA. For Tat ubiquitination, cells were co-transfected with HA-Ub and Tat-Myc. At 36 h post-transfection, MG132 was added to the culture medium to prevent proteasomal degradation. At 48 h post-transfection, cells were washed with cold PBS, harvested, lysed in lysis buffer by sonication, and incubated with protein G agarose beads pre-conjugated with anti-Myc antibody at 4°C overnight. The beads were washed six times with wash buffer. The bead-bound ubiquitinated A3G or Tat proteins were then incubated with USP8-C40 or the catalytic mutant USP8-C40-C786A in an *in vitro* deubiquitination buffer (20 mM Tris-HCl, pH 8.0; 200 mM NaCl; 1 mM EDTA; 10 mM DTT; and 5% glycerol) at 37°C for 3 h, followed by immunoblotting.

### Chemical synthesis of siRNA

The siRNA targeting USP8 and a nonspecific control siRNA were purchased from Sangon Biotech (Shanghai, China). The siUSP8 sense strand sequence was 5′-GGGCCUAUGUACUAUAUAUdTdT-3′.

### Statistical analysis

The data in this study represented three independent experimental replicates and are presented as the mean ± standard deviation (SD). Statistical significance was evaluated using one-way ANOVA or two-tailed Student’s *t*-test. *P < 0.05, **P < 0.01, ***P < 0.001, and ns denotes no significance.

## Data Availability

All data sets generated for this study are within the paper.

## SUPPLEMENTAL MATERIAL

Supplemental material jvi.00598-26-s0001.docx Fig. S1 and S2.

ASM does not own the copyrights to Supplemental Material that may be linked to, or accessed through, an article. The authors have granted ASM a non-exclusive, world-wide license to publish the Supplemental Material files. Please contact the corresponding author directly for reuse.
