# Supplementary material for: Deubiquitinase USP8 inhibits HIV-1 replication by suppressing Tat-mediated viral transactivation
Source: J Virol. 2026 Sep 28;100(10):e00598-26. doi: 10.1128/jvi.00598-26 (PMC13712812; doi:10.1128/jvi.00598-26)
Supplement: Supplementary file 1 [file jvi.00598-26-s0001.docx]

Supplemental Material For

**Deubiquitinase USP8 inhibits HIV-1 replication by suppressing** **Tat-mediated viral transactivation**

Jinsong Yuan^1-2^, Weijing Yang^1-2^, Mingxiu Xu^1-2^, Haobo Hu^1-2^, Shuhan Yang^1-2^, Xiaodong Sun^4^, Wenyan Zhang^1-3^, Chen Huan^1-3^

^1^Department of Infectious Diseases, Infectious Diseases and Pathogen Biology Center, The First Hospital of Jilin University

^2^Institute of Virology and AIDS Research, The First Hospital of Jilin University

^3^Key Laboratory of Organ Regeneration and Transplantation of The Ministry of Education, The First Hospital of Jilin University

^4^Department of Hepatobiliary and Pancreatic Surgery, General Surgery Center, The First Hospital of Jilin University

*Corresponding authors:

Chen Huan, E-mail: [lmsxsml@jlu.edu.cn](mailto:lmsxsml@jlu.edu.cn), Wenyan Zhang, E-mail: [zhangwenyan@jlu.edu.cn](mailto:zhangwenyan@jlu.edu.cn), Xiaodong Sun, E-mail: [sxd@jlu.edu.cn](mailto:sxd@jlu.edu.cn)

This file includes:
Supplemental Fig S1-2

**Fig. S1 USP8 enhances HIV-1 latency in J-Lat cells**

J-Lat-shNC and J-Lat-shUSP8#1, #2 cells were reactivated with TNF-α (20 ng/mL), anti-CD3/CD28 Dynabeads (2 μL per 1 × 10⁵ cells), or PMA (1 μM) for 12, 24, or 36 h. Cells treated with DMSO served as the negative control. GFP-positive cells were detected by flow cytometry. The percentage of GFP-positive cells were quantified using FlowJo.

**Fig. S2 USP8 enhances HIV-1 latency in C11 cells**

C11-shNC and C11-shUSP8#1, #2 cells were reactivated with TNF-α (20 ng/mL), anti-CD3/CD28 Dynabeads (2 μL per 1 × 10⁵ cells), or PMA (1 μM) for 12, 24, or 36 h. Cells treated with DMSO served as the negative control. GFP-positive cells were detected by flow cytometry. The percentage of GFP-positive cells were quantified using FlowJo.
